# Editorial: Identifying and addressing the impact of exposure to maltreatment and experience in children and child serving systems of care

**DOI:** 10.3389/fpsyt.2024.1411792

**Published:** 2024-04-19

**Authors:** Jeanette Scheid, Wynne Morgan, Melissa Kimber

**Affiliations:** ^1^ Department of Psychiatry, Michigan State University, East Lansing, MI, United States; ^2^ Department of Psychiatry, University of Massachusetts Chan Medical School, Worcester, MA, United States; ^3^ Offord Centre for Child Studies, Department of Psychiatry and Behavioural Neurosciences, McMaster University, Hamilton, ON, Canada

**Keywords:** trauma, systems of care, maltreatment, child, adolescent

Child maltreatment is prevalent and contributes to a wide range of emotional and behavioral issues across one’s lifespan. The extant literature on child maltreatment includes its epidemiology, neurobiology, clinical impacts, and related treatments. Over the last several years, increasing attention has been placed on the experiences and impacts of systems of care for children who have been exposed to maltreatment. It was in this context that Frontiers solicited the manuscripts for this Research Topic. In reviewing the work of the 12 teams who submitted manuscripts for this Research Topic, we noted several themes, each of which represents a lesson from the authors and a call for ongoing investigation into understanding how to identify and address risk factors for maltreatment, recognize those affected, and organize systems of care more effectively to provide support. Although specific works are highlighted in each lesson, a careful reading of the manuscripts in this Research Topic reflects each of the themes outlined below.

## Lesson 1

Research must reflect the risks and patterns of maltreatment worldwide. Naved et al. link social determinants, including a more patriarchal culture, to the risk of exposure to violence among boys and girls. Wakuta et al. focus on traumatic interactions in school settings and Zhang et al. explore the impact of parental protection/overcontrol as a risk on the experiences of university students in China. Although not directly examining maltreatment, Au-Yeung et al. describe important work to support the well-being of Indigenous youth.

## Lesson 2

Research must reflect a broader range of traumatic exposures that can contribute to emotional and behavioral problems in children and youth. In the work of Wakuta et al. the impact of teacher-student interactions and later distress is connected, with Zhang et al. linking parent-child relationships, specifically protection and overcontrol to emotional and behavioral health outcomes. Harris et al. describe the complex relationships within families with children who display problematic sexual behaviors; the authors argue for careful consideration and compassion for the experience and well-being of children who exhibit these traits, along with vigilant and comprehensive care for the recipients of these behaviors, when planning effective family-based interventions.

## Lesson 3

Research must reflect the full range of outcomes related to maltreatment exposure. Wakuta et al. examine the phenomenon of Hikikomori, or severe social withdrawal, in relation to traumatic exposures in schools, while in the study by Yu et al. a relationship is observed between co-existing depression and anxiety related to ACES exposure from the UK Biobank data. Thompson and Svendsen explore the characteristics and needs of youth presenting with problematic sexual behaviors. Palmer and Dvir use an ecological systems analysis to review the impact of trauma on individuals with autism spectrum disorder (ASD) and Intellectual Disability (ID).

## Lesson 4

Research and practice must continue to address stress, trauma exposure, and vulnerability to it. Au-Yeung et al. describe early efforts to bring the JoyPop phone application to Indigenous youth, who are at elevated risk for maltreatment. McTavish et al. offer a complementary, clinician-focused discussion and describe a strong case conceptualization, rather than narrower approaches, as a critical frontline tool for serving children and families engaged in child welfare.

## Lesson 5

When addressing complex system issues it is challenging to engage in thinking/working collectively. Joh-Carnella et al. describe the experiences of healthcare providers and child protection teams, identifying effective communication and gaps in collaboration. Howarth et al. describe the challenges faced by teams attempting to create a core set of outcomes to measure the effectiveness of interventions for child-focused domestic abuse. Harris et al. propose using a broader lens than “perpetrator/victim” when attempting to address problematic sexual behaviors in the family setting.

## Lesson 6

Effective listening is an essential element in understanding the lives and experiences of individuals. Au-Yeung et al. work with tribal councils and Indigenous youth so as to evaluate the effectiveness of the applications within JoyPop, and discover that some of their expectations about how youth would respond to certain applications are different from their assumptions. Joh-Carnella et al. use listening methods including qualitative interviews to generate themes related to the collaboration between child protection and healthcare providers. Palmer and Dvir explore the impact of communication challenges faced by children with ASD and IDD (Intellectual and Developmental Disorder) and their impact on identifying trauma exposure. Harris et al. suggest engaging all family members, including the recipients of problematic sexual behaviors, to address family needs and goals.

## Lesson 7

It is possible to accomplish more than one task at a time. McGuier et al. describe the development of identification and referral pathways for post-traumatic stress disorder (PTSD) and related mental health issues within Child Advocacy Centers that had previously focused on investigating allegations of sexual abuse and other maltreatment. Joh-Carnella et al. investigation of the experiences of both child protection teams and healthcare providers points to opportunities for more effective collaboration. The case conceptualization model described by McTavish et al. identifies ways in which system partners can bring their individual expertise to achieve a deeper understanding of families impacted by violence.

## Lesson 8

There is more work to be done. Each of the manuscripts in this Research Topic points to important areas of future inquiry that require robust and sustained research investments. Continued efforts to understand all aspects of the prevention, identification, and impact of child maltreatment remain critical to the health and well-being of individuals across their lifespan.

## Author contributions

JS: Writing – original draft, Writing – review & editing. WM: Writing – review & editing. MK: Writing – review & editing.

